# A Novel Regulatory Cascade Involving BluR, YcgZ, and Lon Controls the Expression of *Escherichia coli* OmpF Porin

**DOI:** 10.3389/fmicb.2017.01148

**Published:** 2017-06-30

**Authors:** Valérie Duval, Kimberly Foster, Jennifer Brewster, Stuart B. Levy

**Affiliations:** Center for Adaptation Genetics and Drug Resistance, Department of Molecular Biology and Microbiology, Tufts University School of Medicine, BostonMA, United States

**Keywords:** regulation, porin, Lon protease, *E. coli*, blue light, temperature

## Abstract

In *Escherichia coli*, OmpF is an important outer membrane protein, which serves as a passive diffusion pore for small compounds including nutrients, antibiotics, and toxic compounds. OmpF expression responds to environmental changes such as temperature, osmolarity, nutrients availability, and toxic compounds via complex regulatory pathways involving transcriptional and post-transcriptional regulation. Our study identified a new regulatory cascade that controls the expression of OmpF porin. This pathway involves BluR, a transcriptional regulator repressing the expression of the *ycgZ-ymgABC* operon. We showed that BluR was responsible for the temperature-dependent regulation of the *ycgZ-ymgABC* operon. Furthermore, our results showed that independent expression of YcgZ led to a decreased activity of the *ompF* promoter, while YmgA, YmgB, and YmgC expression had no effect. We also determined that YcgZ accumulates in the absence of the Lon protease. Thus, mutation in *bluR* leads to de-repression of *ycgZ-ymgABC* transcription. With a second mutation in *lon*, YcgZ protein accumulates to reach levels that do not allow increased expression of OmpF under growth conditions that usually would, i.e., low temperature. With BluR responding to blue-light and temperature, this study sheds a new light on novel signals able to regulate OmpF porin.

## Introduction

The outer membrane of Gram-negative bacteria provides a physical barrier to hydrophobic and hydrophilic compounds including many toxic molecules (Pagès et al., 2008). Embedded in the outer membrane, the porins (outer membrane proteins or OMPs) have multiple functions: allowing the diffusion of small molecules, stabilizing the cell envelope, and acting as receptors for phages and bacteriocins or as virulence factors in pathogenic bacteria (Achouak et al., 2001; Nikaido, 2003; Galdiero et al., 2012). Three major porins are found in abundance in the outer membrane of *Escherichia coli*: OmpA, OmpC, and OmpF (Achouak et al., 2001). With only a small fraction of the porin forming open channels, OmpA seems to be mainly involved in maintaining the shape of the cell (Sugawara and Nikaido, 1994). Conversely, OmpF and OmpC form hydrophilic pores that allow the diffusion of small nutrients and toxic compounds (Chopra and Eccles, 1978; Yoshimura and Nikaido, 1985; Mortimer and Piddock, 1993; Nikaido, 1994, 2003). Although OmpF and OmpC display a similar structure, each porin harbors a unique electrostatic pore potential and consequently a distinct specificity and flow rates for solutes (Cowan et al., 1995; Basle et al., 2003). In this context, OmpF plays a crucial role in the accumulation of small hydrophilic antibiotics such as monoanionic cephalosporins, tetracyclines, and fluoroquinolones (Yoshimura and Nikaido, 1985; Cohen et al., 1989; Mortimer and Piddock, 1993; Duval et al., 2009). In order to respond to changes in environmental conditions, *E. coli* adjusts OmpF and OmpC expression through a complex regulatory network utilizing both transcriptional and translational regulation (for review, see Forst and Inouye, 1988; Pratt et al., 1996; Vogel and Papenfort, 2006). For instance, the osmolarity-dependent transcriptional control of *ompF* and *ompC* is exerted via the EnvZ/OmpR two-component signal transduction system in which EnvZ, an inner membrane histidine protein kinase, senses osmotic signals and transmits them to the transcription factor OmpR (Igo et al., 1989; Egger and Inouye, 1997; Yoshida et al., 2006). High osmolarity leads to lower OmpF levels, while relative expression of OmpC is increased (Forst and Inouye, 1988; Mizuno and Mizushima, 1990; Pratt et al., 1996). In addition, expression of OmpF and OmpC is controlled at the post-transcriptional level by non-translated small RNAs such as MicF, MicC, and RybB (Mizuno et al., 1984; Chen et al., 2004; Gogol et al., 2011).

**FIGURE 1:**
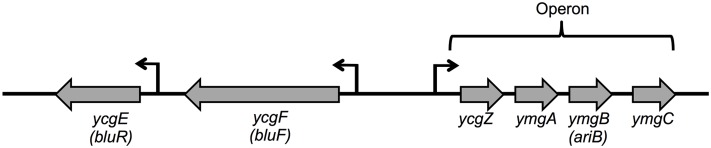
Organization of *ycgZ-ymgABC*, *ycgE*, and *ycgF* ORFs in *Escherichia coli*. The bent arrows indicate the transcription start for each gene. *ycgZ*, *ymgA*, *ymgB*, and *ymgC* genes are expressed as one transcript. *ycgE*, *ycgF*, and *ymgB* have been renamed *bluR*, *bluF*, and *ariB*, respectively.

A major environmental parameter that affects OmpF porin expression is temperature. OmpF is abundant in *E. coli* outer membrane at ambient temperature, while growth at 37°C leads to decreased amount of OmpF. It is assumed that the downregulation of OmpF at body temperature limits the entry of toxic bile salts into the periplasm while the bacterium is in the host’s intestine (Pratt et al., 1996; Nikaido, 2003). In a previous study, we showed that mutation in two genes, *lon* and *bluR* (*ycgE*), prevented the upregulation of OmpF in *E. coli* K-12 grown at ambient temperature (Duval et al., 2009). BluR, a regulator harboring a MerR-like N-terminal domain, has been shown to directly repress the transcription of the adjacent *ycgZ-ymgABC* operon (*ZABC* operon, **Figure 1**; Tschowri et al., 2009). The *lon* locus encodes the Lon protease, an ATP-dependent serine protease involved in the degradation of unstable and misfolded proteins (Tsilibaris et al., 2006; Van Melderen and Aertsen, 2009). Lon also plays a major role in regulating multiple biological processes by controlling the abundance of specific regulatory proteins such as MarA, RcsA, and SulA (Mizusawa and Gottesman, 1983; Torres-Cabassa and Gottesman, 1987; Griffith et al., 2004). With no available studies describing the regulation of OmpF by BluR and Lon, we investigated how BluR and Lon together control the abundance of the OmpF porin. Our study identified the *ZABC* operon as an intermediate in the regulation of OmpF by BluR and Lon. Precisely, we identified YcgZ as a novel repressor of *ompF* expression. Using a transcriptional fusion of the *ompF* promoter with *lacZ* (P*ompF-lacZ*), we showed that YcgZ acted on *ompF* promoter. Finally, our study showed that the amount of YcgZ, when expressed from a plasmid, was amplified and highly stable in a *lon* mutant of *E. coli*, identifying YcgZ as a novel substrate of the Lon protease.

## Materials and Methods

### Bacterial Strains and Growth Conditions

The bacterial strains used in this study are listed in **Table 1**. *E. coli* strains were cultured under agitation in LB medium (tryptone 10 g l^-1^, yeast extract 5 g l^-1^, NaCl 5 g l^-1^). The same medium containing 1.5% agar was used for growing bacteria on plates. Selection of *E. coli* after transformation with pBAD and pMPM vectors was performed using ampicillin 100 μg ml^-1^ and tetracycline 15 μg ml^-1^, respectively. Kanamycin 25 μg ml^-1^ and chloramphenicol 12 μg ml^-1^ was used for selection of chromosomal insertion of *kan* and *cat* genes.

**Table 1:** Bacterial strains and plasmids used in this study.

Strain or plasmid	Genotype/relevant characteristics	Reference or source
**Strains**		
TOP10	*E. coli F- mcrA Δ(mrr-hsdRMS-mcrBC) Φ80lacZΔM15Δ lacX74 recA1 araD139 Δ(araA-leu)7697 galUgalK rpsL endA1 nupG*	Invitrogen, United States
AG100	*E. coli argE3 thi-1 rpsL xyl mtl supE44* λ lysogen	Oethinger et al., 1998
M113R	AG100 *lon3::IS186*	Duval et al., 2009
AGEZ3	AG100 *ycgE::Tn5*	Duval et al., 2009
M113REZ3	AG100 *lon3::IS186 ycgE::Tn5*	Duval et al., 2009
BW25113	F^-^, *Δ(araD-araB)567*, *ΔlacZ4787::rrnB-3*, λ^-^, *rph-1*, *Δ(rhaD-rhaB)568*, *hsdR514*	CGSC (Baba et al., 2006)
JW0419-1	BW25113 Δ*lon-725::kan*	CGSC (Baba et al., 2006)
VD101	BW25113 Δ*bluR::cat*	This study
VD102	BW25113 Δ*lon725::FRT* Δ*bluR::FRT*	This study
VD103	BW25113 *ΔycgZ-ymgABC::cat*	This study
VD104	BW25113 Δ*lon725::FRT* Δ*bluR::FRT ΔycgZ-ymgABC::cat*	This study
VDL25113	BW25113 λ att *ompFp-lacZ* [Amp^R^]	This study
VDL0419	JW0419-1 λ att *ompFp-lacZ* [Amp^R^]	This study
VDL101	VD101 λ att *ompFp-lacZ* [Amp^R^]	This study
VDL102	VD102 λ att *ompFp-lacZ* [Amp^R^]	This study
VDL103	VD103 λ att *ompFp-lacZ* [Amp^R^]	This study
VDL104	VD104 λ att *ompFp-lacZ* [Amp^R^]	This study
**Plasmids**		
pBAD/HisA	Expression cloning vector; [Amp^R^]; pBR322 ori; the *araBAD* promoter initiates the transcription of the target gene	Invitrogen
pDVBZ	pBAD/HisA carrying *ycgZ* nucleotide sequence cloned between *Nco*I and *Pst*I restriction sites; expression of native protein	This study
pDVBZ-XP	pBAD/HisA carrying *ycgZ* nucleotide sequence cloned between *Sac*I and *Pst*I restriction sites; allow the expression of an XPress-tagged YcgZ	This study
pDVBA	pBAD/HisA carrying *ymgA* nucleotide sequence cloned between *Nco*I and *Pst*I restriction sites; expression of native protein	This study
pDVBB	pBAD/HisA carrying *ymgB* nucleotide sequence cloned between *Nco*I and *Pst*I restriction sites; expression of native protein	This study
pDVBC	pBAD/HisA carrying *ymgC* nucleotide sequence cloned between *Nco*I and *Pst*I restriction sites; expression of native protein	This study
pMPM	Expression cloning vector; [Tet^R^]; ori p15A; low copy; the *araBAD* promoter initiates the transcription of the target gene; used in strains carrying λ att *ompFp-lacZ* [Amp^R^]	Mayer, 1995
pDVMZ	pMPM carrying *ycgZ* nucleotide sequence cloned between *Eco*RI and *Xho*I restriction sites; expression of native protein	This study
pDVMA	pMPM carrying *ymgA* nucleotide sequence cloned between *Eco*RI and *Xho*I restriction sites; expression of native protein	This study
pDVMB	pMPM carrying *ymgB* nucleotide sequence cloned between *Eco*RI and *Xho*I restriction sites; expression of native protein	This study
pDVMC	pMPM carrying *ymgC* nucleotide sequence cloned between *Eco*RI and *Pst*I restriction sites; expression of native protein	This study
pDVMbluR	pMPM carrying *bluR* nucleotide sequence, as well as the 200 bp upstream of the start codon and 100 bp downstream of the stop codon; expression of native protein	This study
pRS415	*ori colE1 lacZ* fusion vector, [Amp^R^]	Simons et al., 1987
pDV415O	pRS415 *ompFp-lacZ*	This study

### Plasmids

Expression plasmids used in this study are listed in **Table 1** and were constructed as follows. The nucleotide sequence of *ycgZ*, *ymgA*, *ymgB*, and *ymgC* was amplified by polymerase chain reaction (PCR) using the primers listed in Supplementary Table S1 and *E. coli* AG100 genomic DNA as template. The fragments were cloned into the pBAD/HisA and pMPM vectors using restriction sites indicated in **Table 1**. We constructed the pDVMBluR by PCR amplification of *bluR* nucleotide sequence, the 200 bases upstream of start codon GTG, and the 100 bases downstream of its stop codon TAA using the primers listed in Supplementary Table S1. The PCR fragment was then ligated into the pMPM plasmid using the restriction sites *Eco*RI and *Xho*I, resulting in plasmid pDVMBluR. All nucleotide sequences were verified at the Tufts University Core Facility (Tufts University School of Medicine, Boston, MA, United States).

### Gene Deletion

Targeted deletion of *bluR* and *ycgZ-ymgABC*, and subsequent marker removal were made using the λRed recombinase method previously described (Datsenko and Wanner, 2000). The Flp recombination target (FRT)-flanked chloramphenicol resistance gene (*cat*) has been amplified by PCR from plasmid pKD3 using primers listed in Supplementary Table S1. *bluR-PA/bluR-PB* and *ycgZ-PA/ymgC-PB* primers contain sequences upstream and downstream of *bluR* and of *ycgZ-ymgABC* operon, respectively. The PCR product was gel-purified and concentrated by ethanol precipitation. Transformants carrying the Red helper plasmid pKD46 were then grown in LB medium with 100 μg ml^-1^ ampicillin and 10 mM L-arabinose at 30°C to an optical density at 600 nm (OD_600_) of 0.6 and then made electro-competent. Electroporation was done using 200 ng of PCR product. Chloramphenicol resistant clones were selected on LB agar plates containing chloramphenicol 12 μg ml^-1^. Correct integration of the *cat* gene in the targeted genes was verified by PCR using the primers listed in Supplementary Table S1. Appropriate chloramphenicol resistant clones were subsequently transformed with the pCP20 plasmid and ampicillin resistant clones were selected at 30°C on LB agar plates containing ampicillin 100 μg ml^-1^. The transformants were then colony-purified non-selectively at 42°C on LB agar and then tested for loss of ampicillin and chloramphenicol resistance. Deletions were further verified by PCR using the primers listed in Supplementary Table S1.

### LacZ Transcriptional Fusion and β-Galactosidase Assays

To construct the plasmid pDV415O, amplification of *ompF* promoter (P*ompF*) was carried out by PCR using chromosomal DNA from strain *E. coli* AG100 as template and the primers *ompF1* and *ompF2* listed in Supplementary Table S1. The P*ompF* fragment was 273 bp long (from -273 to +1 relative to the transcription start) and was cloned into the pGEM-T Easy vector (Promega) following the manufacturer instruction. The resulting plasmid was digested with *Eco*RI and *Bam*HI and the fragment corresponding to P*ompF* was ligated to the similarly cut vector pRS415 yielding the plasmids pDV415O. The sequence of the P*ompF-lacZ* fusion in pDV415O was then verified at the Tufts University Core Facility. Insertion of P*ompF-lacZ* into *E. coli* chromosome was realized as followed. Recombination between the pDV415O and λRZ5 (Silhavy et al., 1984; Simons et al., 1987) resulted in a lysate bearing λRZ5 (P*ompF-lacZ*). This was used to infect strains BW25113, a *λ-* and *lac-* strain of *E. coli*. Amp^R^ Lac+ lysogens were selected and purified on LB agar containing ampicillin 20 μg ml^-1^ and X-Gal (5-bromo-4-chloro-3-indolyl-β D-galactopyranoside) 40 μg ml^-1^. Lysates from these lysogens were then used to infect at low multiplicity of infection (MOI = 0.005) strain BW25113 and derivative mutants. Amp^R^ Lac+ lysogens were again isolated and the resulting strains were confirmed by PCR, as previously described (Powell et al., 1994), to have a single copy of the transcriptional fusion located in the λatt site on the chromosome. To assess the β-galactosidase (LacZ) activity, overnight cultures of fresh colonies of *E. coli* carrying a chromosomal λP*ompF-lacZ* fusion were grown in LB medium containing ampicillin 20 μg ml^-1^ and were subsequently diluted to an OD_600_ of 0.05 in identical medium for growth with no antibiotic added. When the OD_600_ reached 0.6, β-galactosidase (LacZ) activity was assayed by first rendered the cells permeable with 0.005% sodium dodecyl sulfate and 0.05% chloroform. LacZ activity was expressed in Miller units as previously described (Miller, 1972). All assays were carried out at least in three independent experiments.

### RNA Isolation

Fresh colonies of *E. coli* strains were grown overnight (14–16 h) in 3 ml of LB medium. The cultures were then diluted to an OD_600_ of 0.05 in 3 ml of identical fresh medium and cells were grown until an OD_600_ of ∼0.6. The total RNAs were isolated from 0.5 ml of culture using the Trizol Reagent (Thermo Fisher Scientific) following the manufacturer protocol. Directly after the preparation of the RNAs, the integrity of the RNAs was evaluated on a bleach gel stained with ethidium bromide (Aranda et al., 2012). The RNAs amount was quantified by absorbance at 260 nm (A260) and purity was evaluated by a A260/A280 ratio >1.9 and a A260/A230 ratio >1.7 using a Nanodrop ND-1000 spectrophotometer (Thermo Fisher Scientific). RNAs were stored for no more than 3 days at -80°C before DNase treatment and cDNA synthesis (see Reverse Transcription and Quantitative PCR Analysis).

### Reverse Transcription and Quantitative PCR Analysis

Two micrograms of purified RNAs were treated using the Turbo^TM^ DNase (Thermo Fisher Scientific) following the manufacturer instructions. A total of 500 ng of RNAs was then used to synthesize cDNA by reverse transcription using the Quantitect Reverse Transcription kit (Qiagen) and following the manufacturer instructions. To control for chromosomal DNA contamination, the reverse transcription step was also performed with reaction mixtures containing no reverse transcriptase and was used as a negative control in subsequent quantitative PCR (qPCR) reactions. Primers used for the qPCR were designed using the online PrimerQuest tool from Integrated DNA Technologies and are listed in Supplementary Table S1. Amplification efficiency and specificity for each set of primers are reported in Supplementary Figure S1. All qPCR reactions were performed using a Roche LightCycler 480 instrument II. The following experimental run protocol was used: UDP activation (50°C for 2 min), denaturation (95°C for 2 min), quantification program repeated 40 times (denaturation at 95°C for 5 s, anneal/extend 60°C for 30 s with a single fluorescence measurement), melting curve program (60–95°C with a heating rate of 0.15°C s^-1^ and a continuous fluorescence measurement). Reactions were carried out in 20 μl with 2 μl of diluted cDNA, 0.4 μl of 10 μM forward primer (0.2 μM final concentration), 0.4 μl of 10 μM reverse primer (0.2 μM final concentration), 7.2 μl H_2_0 and 10 μl of the Power Up SYBR Green Master Mix (Applied Biosystems by Life Technologies). After the reverse transcription step, the cDNA samples were diluted fivefold and used as templates for qPCR amplification of *gapA*, *ompF*, and *ycgZ*. A mix with no cDNA was also prepared (non-template control, NTC) and was run in parallel. NTC wells did either gave a C_T_ > 35 or no C_T_ at all. We quantified the relative expression of *ompF* and *ycgZ* transcripts to the reference gene *gapA*. Using the Pfaffl method (Pfaffl, 2001), we determined the ratio R of a transcript expressed in a sample versus that expressed in the wild type strain grown at 37°C (see C_T_ and calculations in Supplementary Tables S2–S7). *gapA* encodes the glyceraldehydes 3-phosphate deshydrogenase-A.

### Statistical Analysis

**FIGURE 2:**
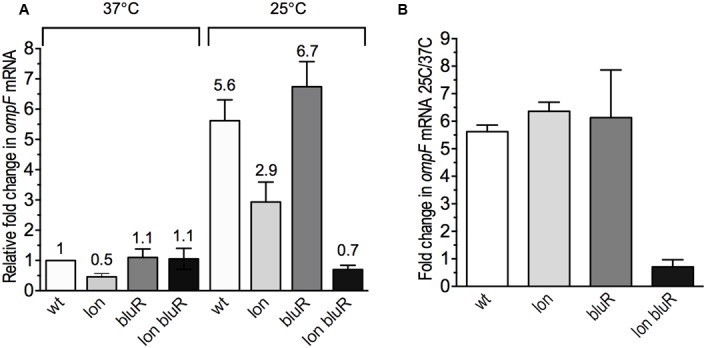
Effect of *lon* and *bluR* mutations on *ompF* mRNA levels in *E. coli* grown at 25 and 37°C. The cells were grown to exponential phase (optical density at 600 nm ∼0.6) in LB medium. Total RNAs were prepared and used to measure the OmpF mRNA levels by RT-qPCR. **(A)** Fold change in OmpF mRNA levels relative to the wild type strain AG100 grown at 37°C. **(B)** Fold change in OmpF mRNA levels for all strains grown at 25 versus 37°C. The numbers represent the means and standard deviations of expression levels from three independent experiments performed in duplicate. AG100, wt; M113R, *lon*; AGEZ3, *bluR*; M113REZ3, *lon bluR*. See Supplementary Tables S2, S3 for details.

We report the average (mean) and the standard deviation (SD) from at least three experimental values. In **Figures 2B** and **4B**, we report the ratio R3 (±SD3) of two numbers R1 ± SD1 and R2 ± SD2, with $\text{R}3=\frac{R2}{R1}\mathrm{and}\frac{\mathrm{SD}3}{R3}=\sqrt{{\left(\frac{\mathrm{SD}1}{R1}\right)}^{2}+{\left(\frac{\mathrm{SD}2}{R2}\right)}^{2}}$. The statistical significance of differences between two averages was determined by a Student’s *t*-test (two independent samples, with two-tailed distribution) using GraphPad Prism software.^1^

### Steady-State Levels of Protein and Stability Assays

Overnight culture of *E. coli* wild type (BW25113) and *lon* (JW0419-1) strains carrying plasmids pBAD/HisA and derivative pDVBZ, pDVBZ-XP, pDVBA, pDVBB, and pDVBC were grown in LB medium in the presence of 100 μg ml^-1^ ampicillin. Fresh identical medium supplemented with L-arabinose was then inoculated to an optical density measured at 600 nm of 0.05 and cells were grown at 37 or 25°C to OD_600_ of 1. Whole cell extracts were prepared for analysis of the steady-state levels of protein. To assess the intracellular stability of YcgZ, 150 μg ml^-1^ chloramphenicol was added to stop the proteins synthesis and the cultures were kept at the indicated temperatures. Samples to be used for preparing whole cell extracts were removed at indicated times (0–60 min). Cell extracts were prepared as follows: 1 ml of culture was centrifuged and the cells pellet suspended in 200 μl of lysis buffer per OD_600_ of 1 [Tris–HCl 10 mM pH 8.0, EDTA 0.5 mM, CaCl_2_ 10 mM, and 1 unit ml^-1^ of DNase (Promega)]. Cells were sonicated on ice two times 20 pulses using a Branson Sonifier 250 and the following parameters: output control = 1 and a duty cycle = 50%. Protein concentration was determined using the Pierce 660 nm Protein Assay Reagent and bovine serum albumin as a standard (Thermo Fisher Scientific). Eight micrograms of proteins were separated on a 16% acrylamide gel in denaturing condition (100 mM Tris, 100 mM Tricine, and 0.1% SDS). The gels were stained with Coomassie Brilliant Blue R-250 (Sigma-Aldrich) or used for western blot analysis.

### Detection of XPress-Tagged YcgZ by Western Blot

After separation on a 16% acrylamide gel, the proteins were electro-transferred to a nitrocellulose membrane (Millipore, Billerica, MA, United States). The membrane was incubated overnight at 4°C in Tris-borate-saline (TBS) buffer supplemented with 3% milk powder. The membrane was then incubated for 2 h at room temperature with monoclonal anti-XPress antibodies (Thermo Fisher Scientific) diluted in TBS (1/6,000). After three 15 min washes with TTBS (TBS supplemented with 0.05% Tween 20), the membrane was incubated for 2 h with alkaline phosphatase-coupled anti-mouse IgG antibodies (Promega) diluted 1/10,000 in TBS followed by three 15 min washes with TTBS and two 5 min washes with TBS. XPress-YcgZ was visualized by adding 5-bromo-4-chloro-3-indolyl phosphate and nitroblue tetrazolium following the manufacturer’s instructions (Promega).

## Results

### *Escherichia coli* Carrying Mutations in both *lon* and *bluR* Failed to Increased OmpF Expression at 25°C

When grown at 37°C, *E. coli* expresses low amount of OmpF in the outer membrane, while decreased temperatures lead to increased levels of the porin (Pratt et al., 1996; Nikaido, 2003). A previous study performed in our laboratory demonstrated that *E. coli* carrying mutations in both *lon* and *bluR* loci resulted in significantly lower amounts of the porin when the cells were grown under low temperature conditions (see protein level of OmpF in Duval et al., 2009). To better characterize the effect of *lon* and *bluR* on *ompF* expression, we performed reverse transcription and qPCR (RT-qPCR) and compared the level of OmpF messenger in wild type *E. coli* AG100 and derivative strains carrying mutations in *lon*, *bluR*, and *lon bluR*. All strains were grown at 37 and 25°C. Our data show that wild type *E. coli* grown at 25°C induces OmpF expression by approximately sixfold when compared to that of wild type *E. coli* grown at 37°C (**Figures 2A,B**). Our data also indicate that a single mutation in *bluR* has no significant effect on OmpF mRNA level, while a *lon* mutation slightly decreases the expression of OmpF at both temperatures (**Figure 2A**). Nevertheless the fold change in OmpF mRNA for both *lon* and *bluR* mutants grown at 25 versus 37°C is similar to that of wild type AG100 (**Figure 2B**). However, a *lon bluR* mutant carrying both mutations fails to increase *ompF* expression when grown at 25°C.

**FIGURE 3:**
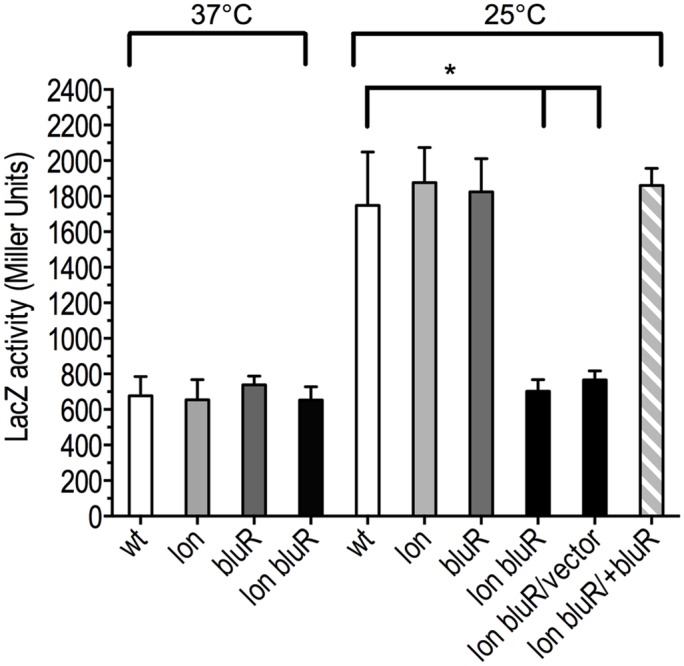
*lon* and *bluR* mutations lead to decreased *ompF* transcription at 25°C. The β-galactosidase activity (Miller units) of a single chromosomal copy of P*ompF-lacZ* was measured for cells grown to exponential phase (optical density at 600 nm ∼0.6) in LB medium at 25 and 37°C. Wild type, VDL25113; *lon*, VDL0419; *bluR*, VDL101; *lon bluR*, VDL102; vector, pMPM; +bluR, pDVMbluR. Numbers represent the means and standard deviations from at least three independent experiments. Statistically significant differences for a mutant compared to wild type are shown as asterisk (^∗^*P* < 0.05).

To further characterize the role of Lon and BluR in regulating OmpF expression, we constructed a transcriptional reporter fusion of *ompF* promoter with *lacZ* (P*ompF-lacZ*). The resulting fusion did not carry *ompF* 5′-UTR affected by MicF (Andersen and Delihas, 1990), but instead carried *lacZ* 5′-UTR. In this case, we prevented post-transcriptional effects on *ompF* 5′-UTR. A single copy of the reporter fusion was then integrated at the att(λ) site of wild type *E. coli* BW25113, a *lac-λ-* strain, and of derivative mutants *lon*, *bluR*, and *lon bluR*. LacZ activity was then assayed in cells grown at 37 and 25°C. Our results from the β-galactosidose assays show that transcription of P*ompF-lacZ* increases by approximately threefold in the wild type strain grown under low temperature conditions (**Figure 3**). In contrast, the *lon bluR* double mutant fails to increase P*ompF-lacZ* expression at 25°C and displays similar P*ompF-lacZ* activities at 25 and 37°C (**Figure 3**). Plasmid mediated expression of *bluR* in the *lon bluR* mutant restores P*ompF-lacZ* levels similar to that of wild type (**Figure 3**). Our data also show that a single mutation in either *lon* or *bluR* has no effect on P*ompF-lacZ* transcription (**Figure 3**). We suspect that the decreased OmpF mRNA level observed for the *lon* mutant (see above) is likely due to a lower stability of the messenger in the absence of the Lon protease since no difference is detected at the transcriptional level.

The simplest model illustrating the effect of both *lon* and *bluR* mutations on *ompF* transcription under the growth conditions used in our experiments implies that BluR represses a locus coding for an intermediate protein that is a substrate of the Lon protease. This intermediate protein would act as a repressor of the *ompF* promoter. In this model, the inactivation of *bluR* leads to an increased transcription of the intermediate locus, and only in the absence or with a reduced activity of Lon, can the intermediate protein accumulate and considerably repress *ompF* at 25°C, a condition that normally permits increased expression of the porin in wild type *E. coli*. In an otherwise direct activation of *ompF* promoter by BluR, deletion of *bluR* alone would lead to decreased *ompF* transcription.

### BluR Controls the Temperature-Dependent Expression of the *ycgZ-ymgABC* Operon

**FIGURE 4:**
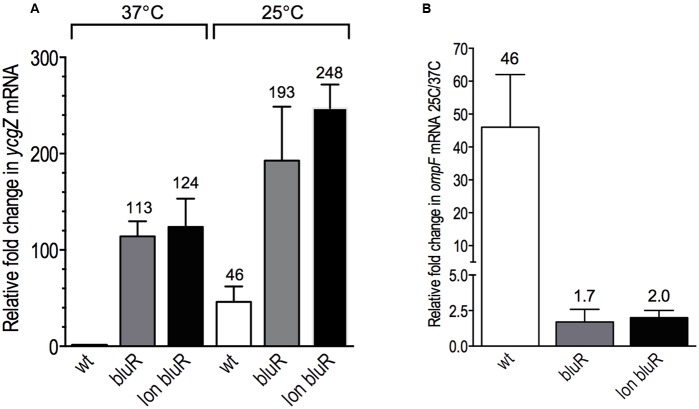
BluR is controlling the temperature-dependent expression of *ycgZ*. **(A)** Fold change in YcgZ mRNA levels relative to the wild type strain AG100 grown at 37°C. **(B)** Fold change in YcgZ mRNA levels for all strains grown at 25 versus 37°C. The cells were grown to exponential phase (optical density at 600 nm ∼0.6) in LB medium. Total RNAs were prepared and used to measure the YcgZ mRNA levels by RT-qPCR. Numbers represent the means and standard deviations of expression levels from three independent experiments performed in duplicate. AG100, wt; AGEZ3, *bluR*; M113REZ3, *lon bluR*. See Supplementary Tables S4, S5 for details.

We subsequently aimed to identify the intermediate locus controlled by BluR and involved in the regulation of *ompF* expression. BluR has been previously described to directly repress the transcription of the *ycgZ-ymgABC* operon (*ZABC*; Tschowri et al., 2012). This operon encodes small proteins of 78–90 amino acid residues involved in biofilm formation through a mechanism that is yet to be determined (Tschowri et al., 2009). Using RT-qPCR, we measured the expression of the *ZABC* operon in AG100 (wt), AGEZ3 (*bluR*), and M113REZ3 (*lon bluR*) derivative strains. When comparing YcgZ mRNA levels at 37 and 25°C for the wild type strain, our results shows a ∼46-fold increased expression of *ycgZ* when the cells were grown at ambient temperature (**Figures 4A,B**). Our data also determine that a *bluR* mutant increases expression of the *ZABC* operon by ∼100- and ∼4-fold in cells grown at 37 and 25°C, respectively (**Figure 4A**), indicating a strong repression of the *ycgZ* promoter by BluR at 37°C. It was previously shown by Tschowri et al. (2009) that BluR–DNA interaction is released in the presence of BluF, a direct antagonist of BluR whose activity is induced by low temperature. We believe that the weaker repression of the *ycgZ* promoter observed at 25°C likely comes from the BluR inactivation by BluF under these growth conditions. At 37°C, BluR repressor is fully active and its deletion leads to a large de-repression of the *ycgZ* promoter. Our results in **Figure 4A** also show that the addition of a *lon* mutation to a *bluR* mutation (*lon bluR*) leads to a slight increased in *ycgZ* mRNA levels when compared to that of *bluR*. For AGEZ3 (*bluR*) and M113REZ3 (*lon bluR*), the low temperature mediated induction is only approximately twofold, overall indicating that *bluR* is mainly responsible for the temperature-dependent expression of *ycgZ*.

### YcgZ Represses *ompF* Transcription

**FIGURE 5:**
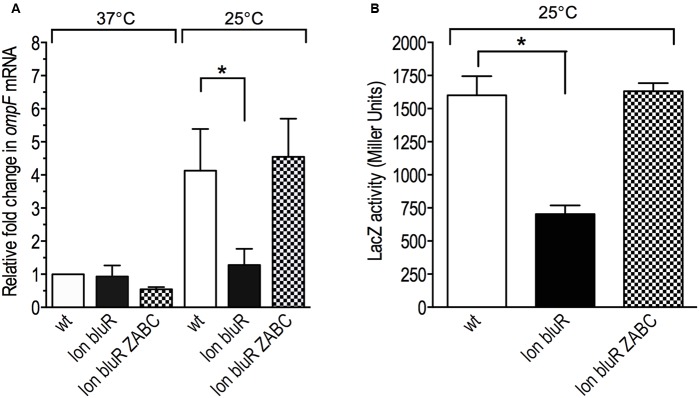
*lon* and *bluR* mutations decrease *ompF* transcription via the *ycgZ-ymgABC* operon. The cells were grown to exponential phase (optical density at 600 nm ∼0.6) in LB medium at 25 and 37°C. Total RNAs were prepared and used to measure the OmpF mRNA levels by RT-qPCR. **(A)** Fold change in OmpF mRNA levels relative to the wild type strain grown at 37°C. **(B)** The β-galactosidase activity (Miller units) of a single chromosomal copy of P*ompF-lacZ* was measured for cells grown to exponential phase in LB medium at 25°C. wt, VDL25113; *lon bluR*, VDL102; *lon bluR ycgZ-ymgABC* (VDL104). The means and standard deviations of at least three independent experiments are shown. Statistically significant differences for a mutant compared to wild type are shown as asterisk (^∗^*P* < 0.05). See Supplementary Table S6, S7 for details.

We then investigated whether the *ZABC* operon, which is de-repressed at 25°C, was involved in the control of *ompF* expression. In our model, if one of the proteins encoded by the *ZABC* operon accumulates in a *lon bluR* strain and represses *ompF*, we expect to see an increased *ompF* expression when the *ZABC* operon is deleted, i.e., in strain *lon bluR ZABC*. Using RT-qPCR, we compared OmpF mRNA levels in wild type, *lon bluR* and *lon bluR ZABC* strains grown at 37 and 25°C. When grown at 37°C, the three strains show similar OmpF mRNA levels (**Figure 5A**). Under low temperature growth conditions, the *lon bluR ZABC* strain expressed OmpF mRNA level similar to that of the wild type strain (**Figure 5A**). We further confirmed that the *ZABC* operon was involved in the control of *ompF* transcription using our P*ompF-lacZ* reporter fusion; **Figure 5B** shows that deletion of the *ZABC* operon in a *lon bluR* background restores LacZ activity similar to that of the wild type. Taken together, our results suggest that the ZABC operon encodes a protein able to repress *ompF* expression. To further identify this repressor, we used a low copy pBAD vector allowing the expression of a target gene from the L-arabinose-dependent promoter *araBAD*. We independently expressed *ycgZ*, *ymgA*, *ymgB*, and *ymgC* in the *lon bluR ZABC* strain and subsequently measured the LacZ activity in the transformants grown at 25°C in the presence of L-arabinose. Our results show that the activity of P*ompF-lacZ* decreases in response to increasing amount of YcgZ, while YmgA, YmgB, and YmgC have no significant effect on P*ompF-lacZ* activity (**Table 2** and see also Supplementary Figure S2). While our results identifies YcgZ as a repressor of the *ompF* promoter and our data show that *ycgZ* expression is high in a *bluR* mutant (**Figure 4**), a *bluR* mutant does not decrease *ompF* transcription (**Figures 2**, **3**). However, a *lon bluR* strain grown at 25°C expresses less *ompF* transcript than the *bluR* mutant. These results strongly suggest that YcgZ is unstable in the presence of Lon under the growth conditions used in our experiments. That a *lon bluR* strain grown at 37°C does not decrease transcription of *ompF* is probably due to growth conditions that inherently lead to a high repression of *ompF* expression. In this case, YcgZ amount may be too low to further repress the *ompF* promoter.

**Table 2:** Expression of P*ompF-lacZ* in *E. coli* VDL104 (*lon bluR ycgZ-ymgABC*) grown to exponential phase in LB medium at 25°C.

Plasmids	L-Arabinose %	LacZ activity	
		Miller units	% Control^a^
pMPM	0	1513 ± 258	
	0.005	1279 ± 201	
	0.05	1066 ± 152	
	0.5	1017 ± 145	
pDVMZ (+*ycgZ*)	0	1195 ± 190	79
	0.005	851 ± 135	66
	0.05	385 ± 49	36
	0.5	225 ± 16	21
pDVMA (+*ymgA*)	0	1221 ± 273	81
	0.5	921 ± 57	91
pDVMB (+*ymgB*)	0	1544 ± 69	102
	0.5	766 ± 140	75
			
pDVMC (+*ymgC*)	0	1324 ± 98	88
	0.5	1068 ± 67	105

*^a^Percentage of LacZ activity compared to the strain carrying the empty vector (pMPM).*

### YcgZ is a Substrate of the Lon Protease

**FIGURE 6:**
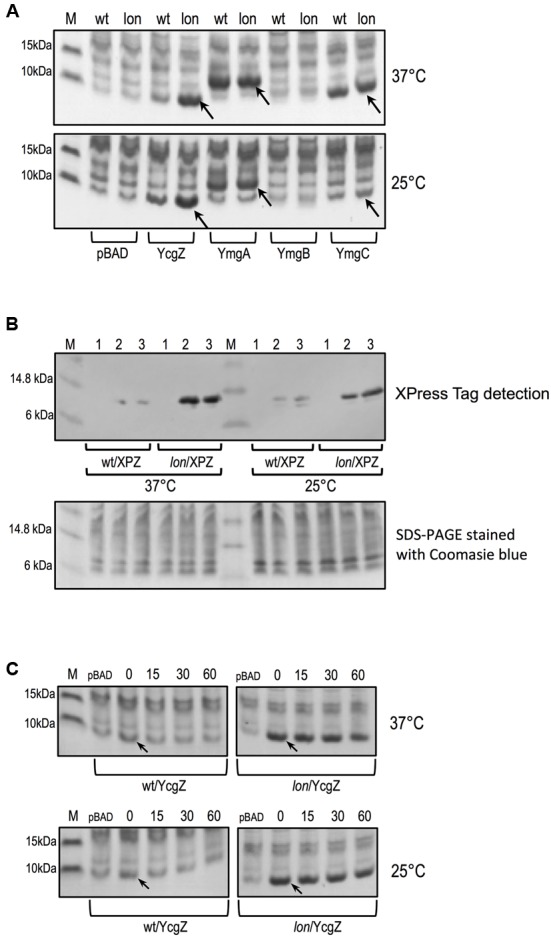
Protein levels of YcgZ, YmgA, YmgB, and YmgC in *E. coli* wild type (BW25113) and *lon* mutant (JW0419-1). **(A)** Steady-state level of native YcgZ, YmgA, YmgB, and YmgC expressed with plasmids pBAD (empty plasmid), pDVBZ (YcgZ), pDVBA (YmgA), pDVBB (YmgB), and pDVBC (YmgC), respectively and 0.05% of L-arabinose. **(B)** Steady-state level of XPress-tagged YcgZ expressed with pDVBZ-XP plasmid and detected by immunoblotting. 1, no L-arabinose; 2, 0.005% L-arabinose; 3, 0.05% L-arabinose. **(C)** Stability of native YcgZ expressed with plasmid pDVBZ. The numbers indicate the time in minutes after addition of 150 μg ml^-1^ chloramphenicol. For all experiments, the target gene was expressed from an *araBAD* promoter using pBAD derivative plasmids. The strains carrying plasmids were grown to an optical density of 1 in the presence of L-arabinose at 37 and 25°C, respectively. Whole cell extracts were then prepared and analyzed by SDS-PAGE and the gels were stained using Coomassie **(A,C)**. The arrow indicates the band corresponding to the expressed protein. Each lane contains 8 μg of proteins. M, Benchmark Protein Ladder.

To evaluate whether YcgZ was a substrate of the Lon protease, we compared YcgZ protein amount in *E. coli* wild type and *lon* mutant. For completion purpose, we also evaluated the stability of YmgA, YmgB, and YmgC. Lacking antibodies that can specifically interact with the proteins, we decided to evaluate the steady-state level of each native protein when overexpressed from an *araBAD* promoter using pBAD derivative plasmids (see **Table 1**). Protein expression was compared between BW25113 (wild type) and JW0419-1 (*lon*) carrying the plasmids and grown in the presence of L-arabinose. Our experiments indicated that the amount of YcgZ was significantly lower when expressed in the wild type strain, while a *lon* mutant accumulated a large amount of YcgZ (**Figure 6A**). Of note, we observed this phenomenon when the cells were grown at both 25 and 37°C. We verified that the lower amount of YcgZ in the wild type was not due to a lesser expression of *ycgZ* by quantifying *ycgZ* messenger levels. RT-qPCR experiments showed that *ycgZ* was similarly expressed in both strains (see Supplementary Table S8), suggesting that the higher level of YcgZ protein observed in the *lon* mutant is likely due to a higher stability of the protein in the absence of Lon. Our data illustrated in **Figure 6A** also shows similar amounts of YmgA and YmgC proteins when overexpressed in the wild type and in the *lon* strains. We could not detect significant amount of YmgB in any of the strains tested even though high level of *ymgB* messenger was measured by RT-qPCR in both strains (data not shown). Using an XPress-tagged YcgZ (XPYcgZ), we confirmed an L-arabinose-dependent accumulation of YcgZ in the *lon* strains, while the protein was barely detectable in the wild type (**Figure 6B**). Additionally, we examined the stability of native YcgZ in the wild type and *lon* strains after stopping the protein synthesis with chloramphenicol and determining the amount of YcgZ in multiple samples taken over a certain period of time (**Figure 6C**). We observed a decline in YcgZ quantity in the wild type strain, while a high amount of the protein was maintained over time in the *lon* mutant, demonstrating a significant stability of YcgZ in the absence of Lon.

**FIGURE 7:**
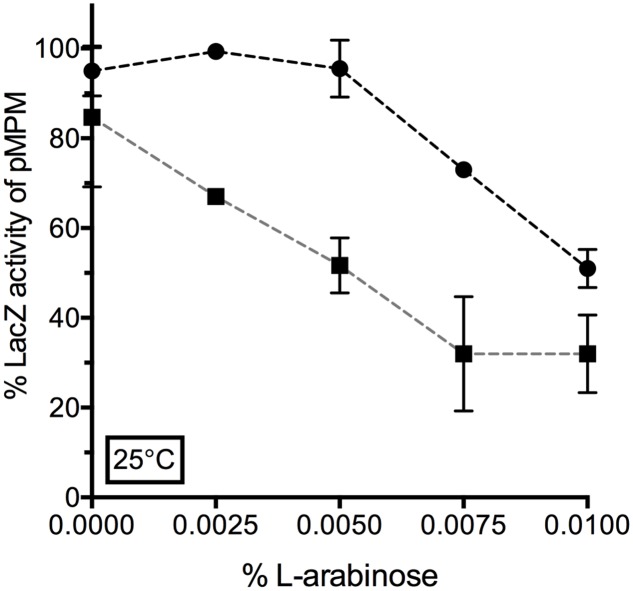
Lon-dependent repression of P*ompF-lacZ* by YcgZ. YcgZ was expressed using the pDVMZ vector in VDL103 (*ycgZ-ymgABC* ●) and VDL104 (*lon bluR ycgZ-ymgABC* ■) strains. Cells were grown at 25°C in LB medium supplemented with increasing concentration of L-arabinose. LacZ activity was measured when the culture reached exponential phase (optical density at 600 nm ∼0.6) and is expressed as a percentage of the LacZ activity measured for the strain carrying the empty pMPM plasmid. The values represent the mean and the standard deviations of a least three independent experiments.

We further aimed to confirm the *lon*-dependent repression of *ompF* promoter by YcgZ. YcgZ was expressed in the *ZABC* and *lon bluR ZABC* mutant strains, both carrying a chromosomal P*ompF-lacZ* fusion and providing both *lon+/lon-* backgrounds. In the absence of *lon*, expression of YcgZ significantly decreased LacZ activity with addition of 0.0025% of L-arabinose (**Figure 7**). In a *lon*+ background, similar repression of *ompF* promoter was reached by increasing the L-arabinose concentration by at least threefold (0.0075%). Ultimately, when a higher concentration of L-arabinose was used (>0.01%), both strains expressed a similar level of LacZ indicating that under those conditions, an abundant level of YcgZ is sufficient to repress *ompF* promoter even in the presence of the Lon protease (**Figure 7**). Of note, the level of *ycgZ* transcript measured when *ycgZ* is expressed from its own promoter is much lower than that expressed from the *araBAD* promoter with concentration of L-arabinose above 0.01% (compared Supplementary Tables S4, S5 with Table S8).

## Discussion

OmpF regulation in response to variable growth conditions provides a great example of bacterial adaptive response to the environment. In this work, we characterize a *lon bluR* mutant of *E. coli* and we show that transcription of *ompF* is impaired when the cells are grown at 25°C. Our data reveal that both *bluR* and *lon bluR* strains expressed high levels of *ycgZ* transcript, confirming a transcriptional repression by BluR on the *ycgZ* promoter. We find that the repression of the *ycgZ* promoter by BluR is strong when the cells are grown at 37°C, while growth at 25°C led to a weaker repression. We also show a high induction of *ycgZ* expression at 25°C, which is essentially mediated by *bluR*. It was previously established that the BluR–DNA interaction is released in the presence of BluF, a direct antagonist of BluR whose activity is induced by low temperature. Inactivation of BluR by BluF consequently led to transcription of the *ycgZ-ymgABC* operon (Tschowri et al., 2009). This effect is further increased upon exposure of BluF to blue-light irradiation in addition to the temperature downshift as blue-light activates BluF through conformational changes (Hasegawa et al., 2006; Nakasone et al., 2010).

While we do not describe the specific mechanism by which YcgZ regulates OmpF expression, our study clearly establishes that expression of YcgZ represses *ompF* transcription. Moreover, our analyses demonstrate a high instability of YcgZ in wild type *E. coli*, while the protein substantially accumulates and remains stable in a *lon* mutant, identifying YcgZ as a substrate of the Lon protease. The high instability of YcgZ in the presence of Lon explains why, even though *ycgZ* expression is large in a *bluR* mutant at 25 and 37°C, *ompF* transcription is not affected. These growth conditions are detrimental to the stability of YcgZ with the activity of Lon being high enough to keep the concentration of YcgZ below the threshold where it can inhibit the *ompF* promoter. In other words, when expressed from its own promoter and under the growth conditions used in our experiments, YcgZ will likely have an effect on the *ompF* promoter only if its stability is increased. Growth at temperatures below 25°C could reduce the proteolytic activity of Lon, leading to a higher level of YcgZ in the cell. For instance, *Yersinia pestis* YmoA is a substrate of the Lon protease. Jackson et al. (2004) found that YmoA stability increases as the growth temperature decreases to become stable at 17°C (half-life >3 h). Alternatively, the interaction of YcgZ with another yet to be discovered protein could protect YcgZ from degradation by Lon. An example of such phenomenon in *E. coli* is illustrated with HU-α and HU-β proteins, two homologous proteins encoded by *hupA* and *hupB*, respectively and which form heterodimers. In the presence of HU-α, HU-β is fairly stable, while in a *hupA* mutant HU-β is degraded by Lon (Bonnefoy et al., 1989); HU-α seems to protect HU-β from degradation by Lon.

How YcgZ, a protein of 78 amino acid residues, acts on *ompF* promoter is still under investigation. YmgB, a small three-helix protein of 88 amino acids encoded by the *ZABC* operon, was shown to display similarity to protein Hha (Lee et al., 2007) and to downregulate curli expression in an RcsB-dependent pathway (Tschowri et al., 2009). It was then proposed that YmgB acts as “connector” of the Rcs phosphorelay. YcgZ could likely work as a small protein connector as well. Interestingly, YmgA and YmgB proteins have been shown to activate colanic acid expression under ambient temperature conditions (16–28°C), while decreasing curli synthesis (Tschowri et al., 2009). The only known role of YcgZ was to somehow alleviate the activity of both YmgA and YmgB.

## Conclusion

**FIGURE 8:**
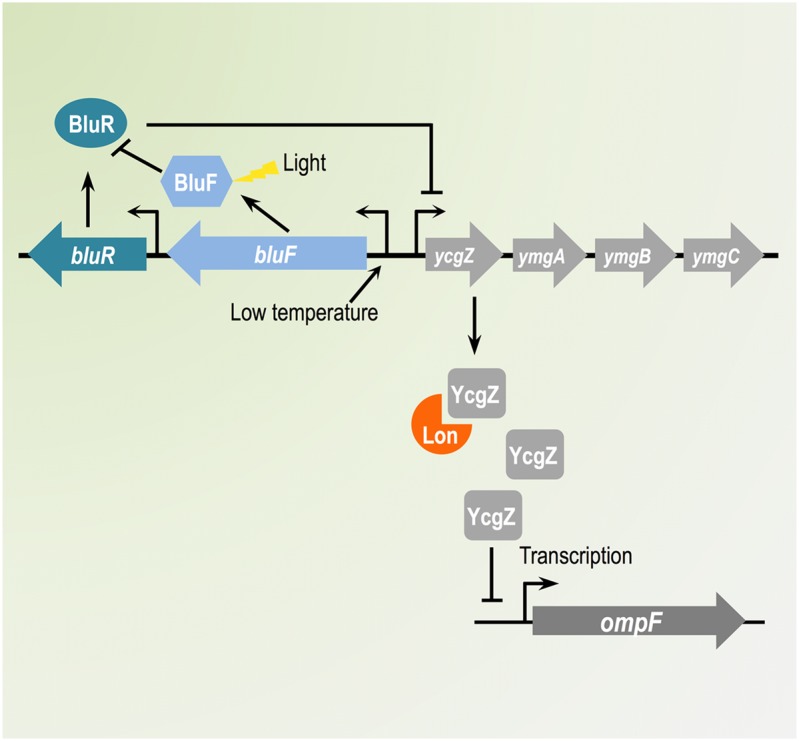
Schematic representation of the regulatory cascade employed by BluR and Lon to control OmpF expression. In *Escherichia coli*, OmpF expression responds to environmental changes via complex regulatory networks. Our report described a novel pathway controlling OmpF synthesis at low temperature. The *ycgZ-ymgABC* operon, which transcription is controlled by the BluR-BluF system, encodes YcgZ, a protein highly unstable in the presence of the Lon protease. Increased amount of YcgZ leads to reduced transcription of *ompF*, consequently decreasing the amount of OmpF porin.

Our study identifies OmpF promoter as a new target regulated by YcgZ, a small pleiotropic regulator, which expression is induced by low temperatures via BluR and is destabilized by the Lon protease. We believe our results shed a new light on novel signals able to regulate OmpF porin expression through complex regulatory pathways (see proposed model in **Figure 8**). The possibility of a more complex regulatory architecture involving other factors not considered in this study is under investigation.

## Author Contributions

VD and SL conceived the study. VD, KF, and JB performed the experiments. VD analyzed data. VD and SL prepared the manuscript and all the authors contributed to preparing the final version of the manuscript. All authors read and approved the final version of the manuscript.

## Conflict of Interest Statement

The authors declare that the research was conducted in the absence of any commercial or financial relationships that could be construed as a potential conflict of interest.

## Footnotes

1 https://www.graphpad.com/scientific-software/prism/
