# Supplementary material for: A Novel Regulatory Cascade Involving BluR, YcgZ, and Lon Controls the Expression of Escherichia coli OmpF Porin
Source: Front Microbiol. 2017 Jun 30;8:1148. doi: 10.3389/fmicb.2017.01148 (PMC5491885; doi:10.3389/fmicb.2017.01148)
Supplement: Supplementary file 1 [file Data_Sheet_1.pdf]

SUPPLEMENTARY MATERIAL.

A novel regulatory cascade involving BluR, YcgZ and Lon controls the expression of Escherichia coli OmpF porin

Valérie Duval, Kimberly Foster, Jennifer Brewster, Stuart B. Levy

Center for Adaptation Genetics and Drug Resistance, Tufts University School of Medicine, Department of Molecular Biology and Microbiology, Boston, MA, USA

Correspondence:

Stuart B. Levy: stuart.levy@tufts.edu

Valerie Duval: valerie.duval@tufts.edu

1. Supplementary Tables.

Table S1.Oligonucleotides used in this study.

Name	Nucleotide sequence 5'-3'	Characteristics
ompF1	GAATTCCATTCTGGATGTCTGAAAGAAGATTTTG	Forward and reverse primers used for amplifying and cloning *ompF* promoter region into the pRS415 vector; EcoRI and BamHI restriction sites
ompF2	GGATCCGTCTGCAGGCATCTTTCCATTCAAAC	
bluR-PA	GTATTATTGGGTCGTGTACAGGCGACGGAGATT TGTGACCGCAAGGAGGAATTGTGGTGTAGGCTG GAGCTGCTTCGAAGTTCCT	Amplification of the Flp recombination target (FRT)-flanked chloramphenicol resistance gene (cat) from plasmid pKD3 containing sequences upstream and downstream of *bluR*
bluR-PB	GTGGGTTCAGATTATAACATTCTGTCTAAGGGG CGGGATAAAGGTGAAATTAATGGGAATTAGCCA TGGTCCATATGAATATC	
bluR-PE	GCACATTCTTTCACATGATTTCAG	Verification of the insertion of *cat* into *bluR* (combination primers *bluR-PE/cat-1* and *bluR-PF/cat-2*)
bluR-PF	GTGAGACTGCGTAGTGTGCACGATC	
ycgZ-PA	GCTATTGTTACTTCACTTAACATTGATTAACATT TTTAACAGAGGCGTAGCATGGTGTAGGCTGGAG CTGCTTCGAAGTTCCT	Amplification of the Flp recombination target (FRT)-flanked chloramphenicol resistance gene (cat) from plasmid pKD3 containing sequences upstream and downstream of the *ycgZ-ymgABC* operon
ymgC-PB	GCATCAGCATGGTGATACAGCTGATGTTTATTCT AAAACCTTACTCAAGTTCTAATGGGAATTAGCC ATGGTCCATATGAATATC	
ycgZ-PE	GTGAGGCGAGAGTAAGACGGTAACAG	Verification of the insertion of *cat* into *ycgZ-ymgABC* (combination primers *ycgZ-PE/cat-1* and *ymgC-PF/cat-2*)
ymgC-PF	GCATCTACAGAGAGCATGGTAGAGAGC	
cat-1	CTTCGAAGCAGCTCCAGCCTACAC	Forward primer in *cat*
cat-2	ACGTGCCGATCAACGTCTCATTTTC	Reverse primer in *cat*
YcgZ-NcoI	AATTAACCATGGGGCATCAAAATTCAGTGACTT TAGATTC	Forward primer used for amplifying *ycgZ*
YcgZ-EcoRI	AAAGAATTCACCATGCATCAAAATTCAGTGACT TTAGATTC	Forward primer used for amplifying ycgZ
YcgZ-XhoI	AAACTCGAGTTATTCAAAAAGCAACCCAATTAG TGC	Reverse primer used for amplifying *ycgZ*
YmgA-NcoI	AATTAACCATGGGGAAGACATCTGATAATGAAC GTATAAAA	Forward primer used for amplifying *ymgA*
YmgA-EcoRI	AAAGAATTCACCATGAAGACATCTGATAATGAA CGTATAAAA	Forward primer used for amplifying *ymgA*
YmgA-XhoI	AAACTCGAGTTAATGTATTCTGTTTATTTTCTTA CCATTG	Reverse primer used for amplifying *ymgA*

YmgB-NcoI	AATTAACCATGGGGCTTGAAGATACTACAATTCATAATGC	Forward primer used for amplifying *ymgB*
YmgB-EcoRI	AAAGAATTCACCATGCTTGAAGATACTACAATTCATAATGC	Forward primer used for amplifying *ymgB*
YmgB-XhoI	AAACTCGAGTTACATATCATCAGCTGTGTATCGCAAC	Reverse primer used for amplifying *ymgB*
YmgC-NcoI	AATTAACCATGGGGAATAATTCAATCCCAGAGAGATTTATTTTTC	Forward primer used for amplifying *ymgC*
YmgC-EcoRI	AAAGAATTCACCATGAATAATTCAATCCCAGAGAGATTTATT	Forward primer used for amplifying *ymgC*
YmgC-PstI	AAACTGCAGCTAAGAGAGCACGGATTCCCTGTCATT	Reverse primer used for amplifying *ymgC*
BluRProm200	CCCCCCGAATTCCAGCACATTCTTTCACATGATTTCAGTAAATC	Amplification and cloning of *bluR* as well as its promoter and terminator regions into the pMPM vector; EcoRI and XhoI restriction sites
BluRTer97	CCCCCCCTCGAGCCCGGTGAGCATTTTGCAACGGACCAG	
GapA-FWS2	CGGTACCGTTGAAGTGAAAGA	Quantitative PCR *gapA*
GapA-RVS2	ACTTCGTCCCATTTCAGGTTAG	
OmpF-FWS2	CCGGTTATGGTCAGTGGGAATA	Quantitative PCR *ompF*
OmpF-RVS2	GCGTATTTAAGACCCGCGAATG	
YcgZ-FWS2	GCATACTCAGCAGGAAACTCT	Quantitative PCR *ycgZ*
YcgZ-RVS2	TGTTCCAGTCGGCAAAGAA	

Table S2: Quantification by RT-qPCR of OmpF transcript expressed in *E. coli* derivative mutants relative to that expressed in *E. coli* AG100 (wt) grown at 37°C (*lon*, M113R; *bluR*, AGEZ3, *lon bluR*, M113REZ3). [(a)] In each experiment, two repeats (replicates) were performed for each cDNA sample tested. Three experiments were performed using cDNA synthesized from different RNAs preparations. We used the average C_T to calculate the ΔC_T for the target gene *ompF* and for the reference gene *gapA* for each experiment. [(b)] Equation $R = \frac{(EompF)^{\Delta CT\ (control-sample)}}{(EgapA)^{\Delta CT\ (control-sample)}}$ was used to measure the ratio of *ompF* transcript expressed in a sample versus that expressed in the AG100 grown at 37°C (control). EompF and EgapA represent the amplification efficiencies obtained for *ompF* and *gapA* primers sets respectively (see Supplementary Figure S1). [(c)] The fold differences represent the means ± SD of the R values obtained in the three experiments and are reported in Figure 2A.

Samples		C_T values						Fold difference [(c)]
		Experiment 1		Experiment 2		Experiment 3		
	Replicate [(a)]	gapA	ompF	gapA	ompF	gapA	ompF	
wt 37°C	1	21.68	20.94	21.81	20.79	21.01	20.20	
	2	21.47	20.87	21.65	20.80	21.14	20.18	
	**Average C_T**	**21.58**	**20.91**	**21.73**	**20.80**	**21.08**	**20.19**	
**$C_{T(wt\ 37°C)}$- $C_{T(wt\ 37°C)}$**		**0**	**0**	**0**	**0**	**0**	**0**	
***R* [(b)]**		***1***		***1***		***1***		***1***
	Replicate	gapA	ompF	gapA	ompF	gapA	ompF	
lon 37°C	1	21.25	21.52	21.91	22.22	21.67	22.41	
	2	21.28	21.48	21.96	21.95	21.56	22.37	
	**Average C_T**	**21.27**	**21.50**	**21.94**	**22.09**	**21.62**	**22.39**	
**$C_{T(wt\ 37°C)}$- $C_{T(lon\ 37°C)}$**		**0.31**	**-0.59**	**-0.21**	**-1.29**	**-0.54**	**-2.2**	
***R* [(b)]**		***0.55***		***0.49***		***0.34***		***0.46 ± 0.11***
	Replicate	gapA	ompF	gapA	ompF	gapA	ompF	
bluR 37°C	1	21.23	20.16	22.17	20.98	21.91	21.41	
	2	21.23	20.10	22.20	21.05	21.89	21.37	
	**Average C_T**	**21.23**	**20.13**	**22.19**	**21.02**	**21.90**	**21.39**	
**$C_{T(wt\ 37°C)}$- $C_{T(bluR\ 37°C)}$**		**0.35**	**0.78**	**-0.46**	**-0.22**	**-0.82**	**-1.2**	
***R* [(b)]**		***1.33***		***1.18***		***0.78***		***1.10 ± 0.28***
	Replicate	gapA	ompF	gapA	ompF	gapA	ompF	
lon bluR 37°C	1	21.50	20.21	21.50	21.12	21.49	20.52	
	2	21.32	20.21	21.46	20.99	21.45	20.52	
	**Average C_T**	**21.41**	**20.21**	**21.48**	**21.06**	**21.47**	**20.52**	
**$C_{T(wt\ 37°C)}$- $C_{T(lon\ bluR\ 37°C)}$**		**0.17**	**0.70**	**0.25**	**-0.26**	**-0.39**	**-0.33**	
***R* [(b)]**		***1.41***		***0.71***		***1.04***		***1.05 ± 0.35***

Table S3: Quantification by RT-qPCR of OmpF transcript expressed in *E. coli* AG100 (wt) and derivative mutants grown at 25°C relative to that expressed in AG100 grown at 37°C (*lon*, M113R; *bluR*, AGEZ3, *lon bluR*, M113REZ3). [(a)] In each experiment, two repeats (replicates) were performed for each cDNA sample tested. Three experiments were performed using cDNA synthesized from three different RNAs preparations. We used the average C_T to calculate the ΔC_T for the target gene *ompF* and for the reference gene *gapA* for each experiment. [(b)] Equation $R = \frac{(EompF)^{\Delta CT\ (control-sample)}}{(EgapA)^{\Delta CT\ (control-sample)}}$ was used to measure the ratio of *ompF* transcript expressed in a sample versus that expressed in the wt grown at 37°C (control). EompF and EgapA are the amplification efficiencies obtained for *ompF* and *gapA* primers sets respectively (Supplementary Figure S1). [(c)] The fold differences represent the means ± SD of the R values obtained in the three experiments and are reported in Figure 2A.

Samples		C_T values						Fold difference [(c)]
		Experiment 1		Experiment 2		Experiment 3		
	Replicate [(a)]	gapA	ompF	gapA	ompF	gapA	ompF	
wt 37°C	1	21.68	20.94	21.81	20.79	21.01	20.20	
	2	21.47	20.87	21.65	20.80	21.14	20.18	
	**Average C_T**	**21.58**	**20.91**	**21.73**	**20.80**	**21.08**	**20.19**	
**$\Delta C_T = C_{T(wt\ 37°C)} - C_{T(wt\ 37°C)}$**		**0**	**0**	**0**	**0**	**0**	**0**	
***R* [(b)]**		***1***		***1***		***1***		***1***
	Replicate	gapA	ompF	gapA	ompF	gapA	ompF	
wt 25°C	1	21.02	17.68	21.15	17.83	20.86	17.18	
	2	21.02	17.70	21.22	17.87	20.90	17.24	
	**Average C_T**	**21.02**	**17.69**	**21.19**	**17.85**	**20.88**	**17.21**	
**$\Delta C_T = C_{T(wt\ 37°C)} - C_{T(wt\ 25°C)}$**		**0.56**	**3.23**	**0.54**	**2.95**	**0.20**	**2.98**	
***R* [(b)]**		***5.77***		***4.87***		***6.22***		***5.62 ± 0.69***
	Replicate	gapA	ompF	gapA	ompF	gapA	ompF	
lon 25°C		20.72	18.50	21.90	19.63	20.67	17.78	
		20.72	18.48	21.94	19.73	20.60	17.75	
	**Average C_T**	**20.72**	**18.49**	**21.92**	**19.68**	**20.64**	**17.77**	
**$\Delta C_T = C_{T(wt\ 37°C)} - C_{T(lon\ 25°C)}$**		**0.86**	**2.42**	**-0.19**	**1.12**	**0.44**	**2.42**	
***R* [(b)]**		***2.76***		***2.38***		***3.66***		***2.93 ± 0.66***
	Replicate	gapA	ompF	gapA	ompF	gapA	ompF	
bluR 25°C	1	21.53	18.03	21.28	17.20	21.71	18.05	
	2	21.58	18.05	21.31	17.33	21.59	18.02	
	**Average C_T**	**21.56**	**18.04**	**21.29**	**17.27**	**21.65**	**18.04**	
**$\Delta C_T = C_{T(wt\ 37°C)} - C_{T(bluR\ 25°C)}$**		**0.02**	**2.87**	**0.44**	**3.53**	**-0.57**	**2.15**	
***R* [(b)]**		***6.53***		***7.60***		***6.04***		***6.74 ± 0.83***
	Replicate	gapA	ompF	gapA	ompF	gapA	ompF	
lon bluR 25°C	1	21.30	20.84	21.15	20.89	21.00	20.77	
	2	21.30	20.87	21.09	20.92	21.01	20.90	
	**Average C_T**	**21.30**	**20.86**	**21.12**	**20.91**	**21.01**	**20.84**	
**$\Delta C_T = C_{T(wt\ 37°C)} - C_{T(lon\ bluR\ 25°C)}$**		**0.28**	**0.05**	**0.61**	**-0.11**	**0.07**	**-0.65**	
***R* [(b)]**		***0.86***		***0.62***		***0.62***		***0.70 ± 0.14***

Table S4: Quantification by RT-qPCR of *ycgZ* transcript expressed in *E. coli* derivative mutants relative to that expressed in *E. coli* AG100 (wt) grown at 37°C (*lon*, M113R; *lon bluR*, M113REZ3). [(a)] In each experiment, two repeats (replicates) were performed for each cDNA sample tested. Three experiments were performed using cDNA synthesized from three different RNAs preparations. We used the average C_T to calculate the ΔC_T for the target gene *ycgZ* and for the reference gene *gapA* for each experiments. [(b)] Equation $R = \frac{(EycgZ)^{\Delta CT\ (control-sample)}}{(EgapA)^{\Delta CT\ (control-sample)}}$ was used to measure the ratio of *ycgZ* transcript expressed in a sample versus that expressed in the wild type grown at 37°C (control). EycgZ and EgapA represent the amplification efficiencies obtained for *ycgZ* and *gapA* primers sets respectively (Supplementary Figure S1). [(c)] The fold differences represent the means ± SD of the R values obtained in the three experiments and are reported in Figure 4A.

Samples		C_T values						Fold difference [(c)]
		Experiment 1		Experiment 2		Experiment 3		
	Replicate [(a)]	gapA	ycgZ	gapA	ycgZ	gapA	ycgZ	
wt 37°C	1	21.68	30.23	21.81	31.09	21.01	29.85	
	2	21.47	30.91	21.65	30.69	21.14	29.71	
	**Average C_T**	**21.58**	**30.57**	**21.73**	**30.89**	**21.08**	**29.78**	
**$\Delta C_T = C_{T(wt\ 37°C)} - C_{T(wt\ 37°C)}$**		**0**		**0**		**0**	**0**	
***R* [(b)]**		***1***		***1***		***1***		***1***
	Replicate	gapA	ycgZ	gapA	ycgZ	gapA	ycgZ	
bluR 37°C	1	21.23	23.06	22.17	25.26	21.90	23.26	
	2	21.23	23.06	22.20	25.85	21.89	23.31	
	**Average C_T**	**21.23**	**23.06**	**22.19**	**25.79**	**21.90**	**23.29**	
**$\Delta C_T = C_{T(wt\ 37°C)} - C_{T(bluR\ 37°C)}$**		**0.35**	**7.51**	**-0.46**	**7.1**	**-0.82**	**6.49**	
***R* [(b)]**		***98.1***		***129.6***		***111.4***		***113.0 ± 15.8***
	Replicate	gapA	ycgZ	gapA	ycgZ	gapA	ycgZ	
lon bluR 37°C	1	21.50	22.53	21.50	23.20	21.49	23.18	
	2	21.32	22.57	21.46	23.06	21.45	23.00	
	**Average C_T**	**21.41**	**22.55**	**21.48**	**23.13**	**21.47**	**23.09**	
**$\Delta C_T = C_{T(wt\ 37°C)} - C_{T(lon\ bluR\ 37°C)}$**		**0.17**	**8.02**	**0.25**	**7.76**	**-0.39**	**6.69**	
***R* [(b)]**		***153.6***		***123.2***		***95.0***		***124.0 ± 29.4***

Table S5: Quantification by RT-qPCR of *ycgZ* transcript expressed in *E. coli* AG100 and derivative mutants grown at 25°C relative to that expressed in AG100 grown at 37°C (*lon*, M113R; *lon bluR*, M113REZ3). [(a)] In each experiment, two repeats (replicates) were performed for each cDNA sample tested. Three experiments were performed using cDNA synthesized from different RNAs preparations. We used the average C_T to calculate the ΔC_T for the target gene *ycgZ* and for the reference gene *gapA* for each experiment. [(b)] Equation $R = \frac{(EycgZ)^{\Delta CT\,(control-sample)}}{(EgapA)^{\Delta CT\,(control-sample)}}$ was used to measure the ratio of *ycgZ* transcript expressed in a sample versus that expressed in the wild type strain grown at 37°C (control). EycgZ and EgapA represent the amplification efficiencies obtained for *ycgZ* and *gapA* primers sets respectively (Figure S1). [(c)] The fold differences represent the means ± SD of the R values obtained in the three experiments and are reported in Figure 4A.

Samples		C_T values						Fold difference [(c)]
		Experiment 1		Experiment 2		Experiment 3		
	Replicate [(a)]	gapA	ycgZ	gapA	ycgZ	gapA	ycgZ	
wt 37°C	1	21.68	30.23	21.81	31.09	21.01	29.85	
	2	21.47	30.91	21.65	30.69	21.14	29.71	
	**Average C_T**	**21.58**	**30.57**	**21.73**	**30.89**	**21.08**	**29.78**	
**$\Delta C_T = C_{T(wt\ 37°C)} - C_{T(wt\ 37°C)}$**		**0**	**0**	**0**	**0**	**0**	**0**	
R [(b)]		***1***		***1***		***1***		***1***
	Replicate	gapA	ycgZ	gapA	ycgZ	gapA	ycgZ	
wt 25°C	1	21.02	24.37	21.15	23.81	20.86	23.97	
	2	21.02	24.31	21.22	23.87	20.90	24.03	
	**Average C_T**	**21.02**	**24.34**	**21.19**	**23.84**	**20.88**	**24.00**	
**$\Delta C_T = C_{T(wt\ 37°C)} - C_{T(wt\ 25°C)}$**		**0.56**	**6.23**	**0.54**	**7.05**	**0.20**	**5.78**	
R [(b)]		***37.6***		***64.6***		***35.8***		***46.0 ± 16.1***
	Replicate	gapA	ycgZ	gapA	ycgZ	gapA	ycgZ	
bluR 25°C	1	21.53	22.72	21.28	21.73	21.71	22.30	
	2	21.58	22.71	21.31	21.84	21.59	22.48	
	**Average C_T**	**21.56**	**22.72**	**21.29**	**21.79**	**21.65**	**22.39**	
**$\Delta C_T = C_{T(wt\ 37°C)} - C_{T(bluR\ 25°C)}$**		**0.02**	**7.85**	**0.44**	**9.10**	**-0.57**	**7.39**	
R [(b)]		***152.5***		***256.7***		***168.8***		***192.7 ± 56.1***
	Replicate	gapA	ycgZ	gapA	ycgZ	gapA	ycgZ	
lon bluR 25°C	1	21.30	21.59	21.15	21.52	21.00	21.34	
	2	21.30	21.60	21.09	21.66	21.01	21.27	
	**Average C_T**	**21.30**	**21.60**	**21.12**	**21.59**	**21.01**	**21.31**	
**$\Delta C_T = C_{T(wt\ 37°C)} - C_{T(lon\ bluR\ 25°C)}$**		**0.28**	**8.97**	**0.61**	**9.30**	**0.07**	**8.47**	
R [(b)]		***262.5***		***260.3***		***219.1***		***248.0 ± 25.1***

Table S6: Quantification by RT-qPCR of OmpF transcript expressed in *E. coli* derivative mutants relative to that expressed in *E. coli* BW25113 (wt) grown at 37°C (*lon*, JW0419-1; *lon bluR*, VD102; *lon bluR ZABC*, VD104). [a] In each experiment, two repeats (replicates) were performed for each cDNA sample tested. Three experiments were performed using cDNA synthesized from different RNAs preparations. We used the average C_T to calculate the ΔC_T for the target gene *ompF* and for the reference gene *gapA* for each experiment. [b] Equation $R = \frac{(EompF)^{\Delta CT\ (control-sample)}}{(EgapA)^{\Delta CT\ (control-sample)}}$ was used to measure the ratio of *ompF* transcript expressed in a sample versus that expressed in the BW25113 grown at 37°C (control). EompF and EgapA represent the amplification efficiencies obtained for *ompF* and *gapA* primers sets respectively (Figure S1). [c] The fold differences represent the means ± SD of the R values obtained in the three experiments and are reported in Figure 5A.

Samples		C_T values						Fold difference [c]
		Experiment 1		Experiment 2		Experiment 3		
	Replicate [a]	gapA	ompF	gapA	ompF	gapA	ompF	
wt 37°C	1	20.48	19.55	20.09	19.23	20.31	20.52	
	2	20.31	19.53	20.08	19.28	20.93	20.05	
	**Average C_T**	**20.40**	**19.54**	**20.09**	**19.26**	**20.62**	**20.29**	
**$\Delta C_T = C_{T(wt\ 37°C)} - C_{T(wt\ 37°C)}$**		**0**	**0**	**0**	**0**	**0**	**0**	
***R* [b]**		***1***		***1***		***1***		***1***
	Replicate	gapA	ompF	gapA	ompF	gapA	ompF	
lon bluR 37°C	1	20.30	20.11	20.79	20.14	21.13	20.34	
	2	20.21	20.11	20.79	20.18	21.10	20.45	
	**Average C_T**	**20.26**	**20.11**	**20.79**	**20.16**	**21.12**	**20.40**	
**$\Delta C_T = C_{T(wt\ 37°C)} - C_{T(lon\ bluR\ 37°C)}$**		**0.14**	**-0.57**	**-0.70**	**-0.90**	**-0.50**	**-0.11**	
***R* [b]**		***0.63***		***0.87***		***1.29***		***0.93 ± 0.33***
	Replicate	gapA	ompF	gapA	ompF	gapA	ompF	
lon bluR ZABC 37°C	1	19.93	20.03	20.54	20.81	19.63	20.83	
	2	19.93	20.03	20.50	20.73	19.61	21.18	
	**Average C_T**	**19.93**	**20.03**	**20.52**	**20.77**	**19.62**	**21.01**	
**$\Delta C_T = C_{T(wt\ 37°C)} - C_{T(lon\ bluR\ ZABC\ 37°C)}$**		**0.47**	**-0.49**	**-0.43**	**-1.51**	**1.00**	**-0.72**	
***R* [b]**		***0.53***		***0.49***		***0.32***		***0.45 ± 0.11***

Table S7: Quantification by RT-qPCR of OmpF transcript expressed in *E. coli* BW25113 (wt) and derivative mutants grown at 25°C relative to that expressed in BW25113 grown at 37°C (*lon*, JW0419-1; *lon bluR*, VD102; *lon bluR ZABC*, VD104). [(a)] In each experiment, two repeats (replicates) were performed for each cDNA sample tested. Three experiments were performed using cDNA synthesized from different RNAs preparations. We used the average C_T to calculate the ΔC_T for the target gene *ompF* and for the reference gene *gapA* in each experiment. [(b)] Equation $R = \frac{(EompF)^{\Delta CT\ (control-sample)}}{(EgapA)^{\Delta CT\ (control-sample)}}$ was used to measure the ratio of *ompF* transcript expressed in a sample versus that expressed in the BW25113 grown at 37°C (control). EompF and EgapA represent the amplification efficiencies obtained for *ompF* and *gapA* primers sets respectively (Figure S1). [(c)] The fold differences represent the means ± SD of the R values obtained in the three experiments and are reported in Figure 5A.

Samples		C_T values						Fold difference [(c)]
		Experiment 1		Experiment 2		Experiment 3		
	Replicate [(a)]	gapA	ompF	gapA	ompF	gapA	ompF	
wt 37°C	1	20.48	19.55	20.09	19.23	20.31	20.52	
	2	20.31	19.53	20.08	19.28	20.93	20.05	
	**Average C_T**	**20.40**	**19.54**	**20.09**	**19.26**	**20.62**	**20.29**	
**$\Delta C_T = C_{T(wt\ 37°C)} - C_{T(wt\ 37°C)}$**		**0**	**0**	**0**	**0**	**0**	**0**	
***R* [(b)]**		***1***		***1***		***1***		***1***
	Replicate	gapA	ompF	gapA	ompF	gapA	ompF	
wt 25°C	1	20.32	17.60	20.08	17.55	20.32	17.10	
	2	20.27	17.45	20.14	17.39	20.45	17.80	
	**Average C_T**	**20.30**	**17.53**	**20.11**	**17.47**	**20.39**	**17.45**	
**$\Delta C_T = C_{T(wt\ 37°C)} - C_{T(wt\ 25°C)}$**		**0.10**	**2.01**	**-0.02**	**1.79**	**0.23**	**2.84**	
***R* [(b)]**		***3.50***		***3.29***		***5.55***		***4.11 ± 1.26***
	Replicate	gapA	ompF	gapA	ompF	gapA	ompF	
lon bluR 25°C		19.69	19.05	20.20	19.06	20.33	19.16	
		19.60	19.06	20.24	19.14	20.49	19.20	
	**Average C_T**	**19.65**	**19.06**	**20.22**	**19.10**	**20.41**	**19.18**	
**$\Delta C_T = C_{T(wt\ 37°C)} - C_{T(lon\ bluR\ 25°C)}$**		**0.75**	**0.48**	**-0.13**	**0.16**	**0.21**	**1.11**	
***R* [(b)]**		***0.83***		***1.20***		***1.80***		***1.28 ± 0.49***
	Replicate	gapA	ompF	gapA	ompF	gapA	ompF	
lon bluR ZABC	1	20.14	17.30	20.17	17.15	20.74	17.80	
25°C	2	20.19	17.46	20.27	17.19	20.78	17.72	
	**Average C_T**	**20.17**	**17.38**	**20.22**	**17.17**	**20.76**	**17.76**	
**$\Delta C_T = C_{T(wt\ 37°C)} - C_{T(lon\ bluR\ ZABC\ 25°C)}$**		**0.23**	**2.16**	**-0.13**	**2.09**	**-0.14**	**2.53**	
***R* [(b)]**		***3.54***		***4.30***		***5.80***		***4.55 ± 1.15***

Table S8: Quantification by RT-qPCR of *ycgZ* transcript expressed in *E. coli* BW25113 (wt) and JW0419-1 (lon) when expressed using pDVBZ vector and 0.05% of L-arabinose (wt/Z and lon/Z). [(a)] Two repeats (replicates) were performed for each cDNA sample tested. One experiment was performed with RNAs prepared from the same *E. coli* cultures used to prepare the protein samples shown in Figure 6A. We used the average C_T to calculate the ΔC_T for the target gene *ycgZ* and for the reference gene *gapA*. [(b)] Equation $R = \frac{(EycgZ)^{\Delta CT\ (control-sample)}}{(EgapA)^{\Delta CT\ (control-sample)}}$ was used to measure the ratio of *ycgZ* transcript expressed in a sample versus that expressed in the wild type carrying the empty plasmid pBAD/HisA grown at 37°C in the presence of 0.05% of L-arabinose (control). EycgZ and EgapA represent the amplification efficiencies obtained for *ycgZ* and *gapA* primers sets respectively (Figure S1).

Samples		C_T values	
		Experiment 1	
	Replicate [(a)]	gapA	ycgZ
wt/pBAD-HisA 37°C	1	21.10	28.77
	2	20.97	28.99
	**Average C_T**	**21.04**	**28.88**
**$\Delta C_T = C_{T(wt/pBAD\ 37°C)} - C_{T(wt/pBAD\ 37°C)}$**		**0**	**0**
R [(b)]		***1***	
	Replicate	gapA	ycgZ
wt/pDVBZ 37°C	1	21.54	13.85
	2	21.83	13.96
	**Average C_T**	**21.69**	**13.91**
**$\Delta C_T = C_{T(wt/pBAD\ 37°C)} - C_{T(wt/Z\ 37°C)}$**		**-0.65**	**14.97**
R [(b)]		***22986***	
	Replicate	gapA	ycgZ
wt/pDVBZ 25°C		20.11	15.18
		20.82	15.28
	**Average C_T**	**20.47**	**15.23**
**$\Delta C_T = C_{T(wt/pBAD\ 37°C)} - C_{T(wt/Z\ 25°C)}$**		**0.57**	**13.65**
R [(b)]		***4362***	
	Replicate	gapA	ycgZ
lon/pBAD-HisA 37C	1	21.44	28.13
	2	21.16	28.25
	**Average C_T**	**21.30**	**28.19**
**$\Delta C_T = C_{T(wt/pBAD\ 37°C)} - C_{T(lon/pBAD\ 37°C)}$**		**-0.26**	**0.69**
R [(b)]		***1.85***	
	Replicate	gapA	ycgZ
lon/pDVBZ 37°C	1	21.20	13.32
	2	21.56	13.63
	**Average C_T**	**21.39**	**13.48**
**$\Delta C_T = C_{T(wt/pBAD\ 37°C)} - C_{T(lon/Z\ 37°C)}$**		**-0.35**	**15.40**
R [(b)]		***24792***	
	Replicate		
lon/pDVBZ 25°C	1	21.28	15.30
	2	21.12	15.42
	**Average C_T**	**21.20**	**15.36**
**$\Delta C_T = C_{T(wt/pBAD\ 37°C)} - C_{T(lon/Z\ 25°C)}$**		**-0.16**	**13.52**
R [(b)]		***6534***	

2. Supplementary Figures.

A.

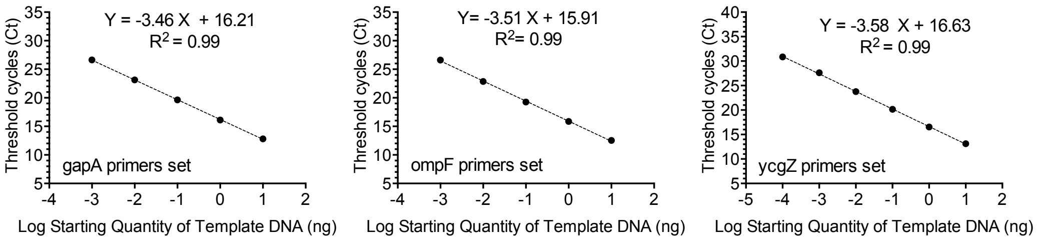

B.

Primers set	Slope	Efficiency (E)	% Efficiency
gapA	-3.46 ± 0.02	1.95	95%
ompF	-3.51 ± 0.05	1.93	93%
ycgZ	-3.58 ± 0.04	1.90	90%

C.

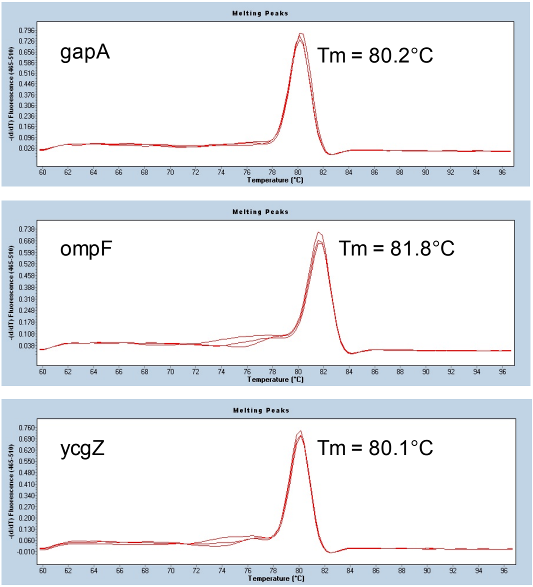

Figure S1. Amplification efficiency and melting curve analysis of each set of primers used for the qPCR experiments. (A) Determination of the amplification efficiencies. The curves were constructed by plotting the log of the starting quantity of *E. coli* AG100 genomic DNA against the C_T value obtained during PCR amplification. The equation for the linear regression as well as the coefficient of determination (R^2) were calculated using GraphPad Prims 6 (www.graphpad.com) and are shown above each graph (n=3). (B) Amplification efficiency (E= $10^{-1/slope}$) and percentage of efficiency (%E= (E-1) x 100%) measured for each set of primers. (C) Melting curves analysis recovered after qPCR runs using the lowest input of cDNA template. The negative first derivative of the change in fluorescence is plotted as a function of temperature. The numbers indicate the melting temperature of single specific products.

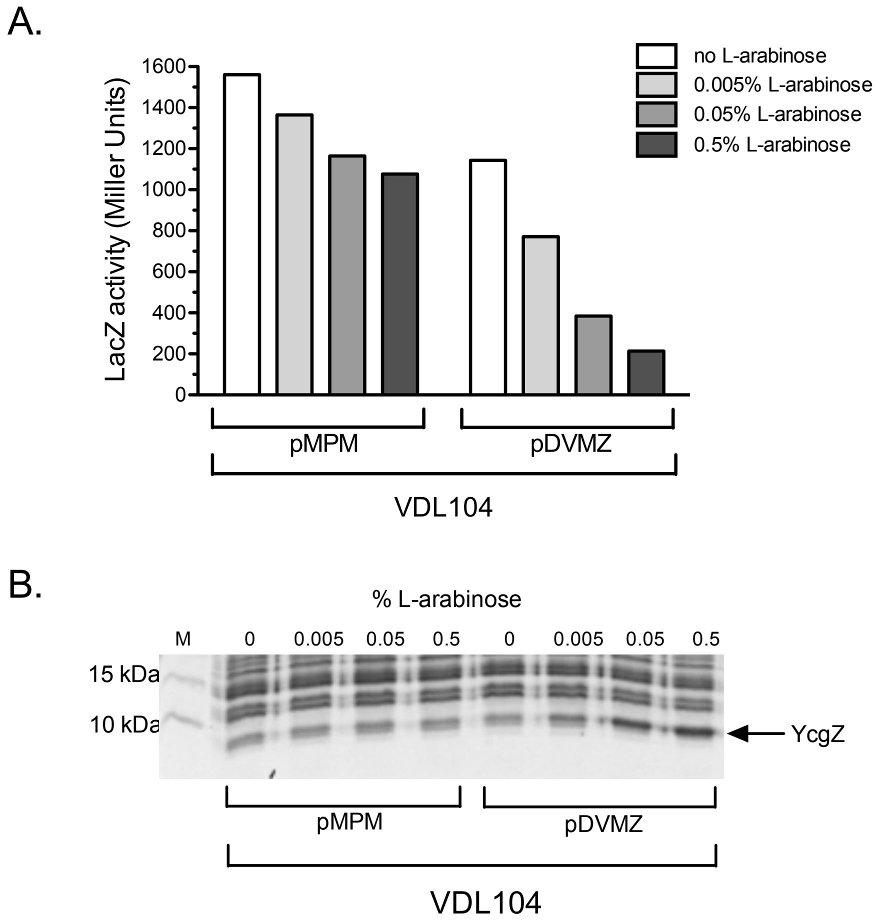

Figure S2. Repression of OmpF promoter by YcgZ. (A) L-arabinose-dependent activity of P*ompF-lacZ* in VDL104 (*lon bluR ycgZ-ymgABC*) when YcgZ is expressed using pDVMZ plasmid. (B) SDS-PAGE using a 16% acrylamide gel showing a L-arabinose-dependent increased in YcgZ protein in strain VDL104/pDVMZ. M, Benchmark Protein Ladder.
